# Thermal Behavior of the Time-Dependent Radiative Flow of Water-Based CNTs/Au Nanoparticles Past a Riga Plate with Entropy Optimization and Multiple Slip Conditions

**DOI:** 10.3390/e25010076

**Published:** 2022-12-30

**Authors:** K. Rajupillai, Nazek Alessa, S. Eswaramoorthi, Karuppusamy Loganathan

**Affiliations:** 1 Department of Mathematics, Government College of Technology, Coimbatore 641013, Tamil Nadu, India; 2 Department of Mathematical Sciences, College of Sciences, Princess Nourah bint Abdulrahman University, P.O. Box 84428, Riyadh 11671, Saudi Arabia; 3 Department of Mathematics, Dr. N.G.P. Arts and Science College, Coimbatore 641048, Tamil Nadu, India; 4 Department of Mathematics and Statistics, Manipal University Jaipur, Jaipur 303007, Rajasthan, India

**Keywords:** SWCNTs, MWCNTs, HAM, radiation, heat consumption/generation

## Abstract

This communication deliberates the time-reliant and Darcy–Forchheimer flow of water-based CNTs/gold nanoparticles past a Riga plate. In addition, nonlinear radiation, heat consumption and multiple slip conditions are considered. Entropy generation is computed through various flow parameters. A suitable transformation with symmetry variables is invoked to remodel the governing mathematical flow models into the ODE equations. The homotopy analysis scheme and MATLAB bvp4c method are imposed to solve the reduced ODE equations analytically and numerically. The impact of sundry flow variables on nanofluid velocity, nanofluid temperature, skin friction coefficient, local Nusselt number, entropy profile and Bejan number are computed and analyzed through graphs and tables. It is found that the nanofluid velocity is reduced by greater porosity and slip factors. The thickness of the thermal boundary layer increases with increasing radiation, temperature ratio, and heat consumption/generation parameters. The surface drag force is reduced when there is a higher Forchheimer number, unsteadiness parameter and porosity parameter. The amount of entropy created is proportional to the radiation parameter, porosity parameter and Reynolds number. The Bejan number profile increases with radiation parameter, heat consumption/generation parameter and the Forchheimer number.

## 1. Introduction

Due to their intriguing thermal transport features, nanofluids have fascinated a large number of researchers in recent decades. The customary heat transmittal fluids, such as water, ethylene glycol and oil, have lesser fundamental heat transport properties. In order to boost the thermal conductivity of customary heat transmittal fluids, nanofluids (the homogeneous mingling of solid nanoparticles of dimension 1–100 nm in customary heat transmittal fluids) are often used. When it comes to heat conductivity, carbon nanotubes (CNTs) are far and away the best nano-element, with a wonderful capability of conducting heat. CNTs are rolled-up graphene sheets with the simplest atomic bonding arrangement. Because of their nanoscale size, they exhibit exceptional structural and mechanical characteristics and excellent electrical and thermal conductivity. The flow of CNTs upon a stretchy sheet with suction was probed by Saleh et al. [1]. They detected that MWCNTs have a higher temperature than the SWCNTs. Anuar et al. [2] found that kerosene-based CNTs have a larger HT gradient than water-based CNTs for the flow of CNTs past a moving plate with stability analysis. The 2D flow of water-based CNTs past a curved surface was numerically computed by Hayat et al. [3]. Kamali and Binesh [4] detected that the addition of MWCNTs leads to improving the HT coefficient for the problem of water-based CNTs of non-Newtonian nanofluids. The heat transmission of water-based SWCNTs inside a heated circular pipe was numerically addressed by Saeed et al. [5]. They concluded that the maximum improvement of the Nusselt number was attained at a 0.05% volume fraction of SWCNTs. Hussain et al. [6] proved that water-based SWCNTs generate a larger HT rate than water-based MWCNTs on their study of the flow of CNTs between two spinning plates. The 3D DF flow of CNTs in a heated rotating frame was explored by Hayat et al. [7]. They found that NPVF leads to improve the nanofluid temperature. The flow of graphene-based nanofluid past a cylinder was investigated by Ghani et al. [8]. They found that increased heat flux was obtained in graphene–water than the SWCNT–water.

Magnetic fields are essential in several scientific, technical and industrial operations. However, commonly used electrically conducting fluids, such as plasma, electrolytes and liquid metals, are poor electrical conductors. Enhancing conductivity is a challenging endeavor for researchers. Incorporating an external agent is one of the most straightforward approaches to enhancing electrical conductivity. A Riga plate is an example of an external material that may be used to boost the electrical conductivity of fluids. This plate is made by combining an implanted magnetic bar or magnets with electrodes that are arranged in alternating positions. The flow of chemically reacting second-grade fluid over a heated Riga plate was presented by Rasool et al. [9]. They demonstrated that the changed Hartmann number leads to decrease the fluid temperature. Vishnu Ganesh et al. [10] proved that the temperature gradient decreases when improving the modified Hartmann number for their problem of the flow of a nanofluid past a Riga plate. The slip impact of nanofluid flow through a Riga plate was addressed by Nadeem Abbas et al. [11]. They noticed that the SFC decreases when enriching the modified Hartmann number. Hanumesh Vaidya et al. [12] identified that a nanofluid’s temperature drops when increasing the modified Hartmann number in a study of the impact of mixed convection of nanofluid flow over a Riga plate. The effect of a heat source/sink on a flow of Maxwell nanofluid past a heated Riga plate was analyzed by Ramesh et al. [13]. Madhukesh et al. [14] deliberated the salient features of slip effects of water-based SWCNTs past a Riga plate with microorganisms. They noticed that the density of motile microorganism decreased when the modified Hartmann number was increased.

In recent years, many scientists have been willing to scrutinize the radiation impact because of its widespread usage in various fields such as power plants, atomic plants, glass making, projectiles, propulsion devices, space exploration and gas turbines. The linearized Rosseland approximation is widely used; however, it only works when the fluid and surrounding temperatures are close. However, in many industrial situations, the difference between the fluid and surrounding temperature is high, and this method is insufficient. In this situation, a non-linear Rosseland approximation is used. The non-linear radiative 2D flow of nanofluid past a Riga plate was inspected by Waqas et al. [15]. They noticed that the nanofluid temperature increases with higher temperature ratio parameter. Ghasemi et al. [16] noted that a thicker thermal boundary layer occurs for larger values of thermal radiation for the problem of radiative flow of nanofluid flow over an SS with magnetic impact. The non-linear radiative 3D flow of nanofluid past a Riga plate was presented by Abdul Hakeem et al. [17], and they uncovered that increased temperature ratio improved the HT rate. Gautam et al. [18] detected that the thicker thermal boundary layer occurs in a Maxwell fluid than in a Casson fluid when changing the temperature ratio parameter for the problem of MHD flow of Maxwell/Casson fluid flow past an SS with radiation. The MHD flow of a tangent hyperbolic hybrid nanofluid past a heated SS was examined by Rashid et al. [19]. They noticed that a higher temperature ratio leads to improve the nanofluid temperature. Eswaramoorthi et al. [20] achieved that the heat transfer gradient is enhanced with increasing temperature ratio for 3D DF flow of CNTs past a Riga plate with glycerin as a base fluid.

Entropy production is a physical phenomenon inherent in all heat-transmission configurations linked to thermodynamic irreversibility. Any thermal system with a high entropy production rate has its usable work destroyed and its efficiency drastically reduced. The term “entropy generation minimization” (EGM) was first introduced by Bejan [21,22] to quantify and optimize the creation of order from chaos in a wide range of processes, including cryogenics, heat transfer, heat exchangers, storage, turbomachinery and electronic cooling. The entropy optimization of a water-based nanofluid past an SS with heat consumption was addressed by Eswaramoorthi et al. [23]. They used Cu and Ag nanoparticles in their study and found that the Bejan number increases when the radiation parameter raises. Dadheech et al. [24] found that the Brinkman number improves the entropy profile for their problem of radiative Williamson fluid flow in a porous stretching surface. The entropy generation of a steady, MHD Carreau fluid flow past a microchannel with radiation was deliberated by Srinivas Reedy et al. [25]. They proved that the Biot number creates more entropy generation and a high Bejan number. The flow of MHD viscous fluid past a porous microchannel with entropy generation was addressed by Abbas et al. [26]. They ascertained that a higher entropy generation rate is obtained for stronger magnetic field and injection parameters. Ibrahim et al. [27] proved that the higher values of Prandtl number decrease the entropy profile for the analysis of the 3D mixed convective flow of a couple-stress nanofluid. Entropy minimization of MHD flow of a Carreau–Yasuda fluid past a SS was illustrated by Khan et al. [28].

According to the aforementioned literature survey, the variations of entropy and HT of water-based CNTs with non-linear radiation past a Riga plate have not been fully investigated. Therefore, our study’s primary goal is to fill this information gap. The primary objective of this body of study is to investigate the impact of time-dependent DF flow of water-based CNTs/gold nanoparticles past a Riga plate with non-linear thermal radiation and heat consumption. The effects of a variety of different factors on the skin friction coefficient, local Nusselt number, entropy generation and the Bejan number are discussed using tabular and graphical representations. Our results are unique and can be used in many industrial areas, including the design of electrical equipment, solar collectors, storage of harmful heat exchangers, freezing, and growing of crystal and glass.

## 2. Mathematical Formulation

**Figure 1:**
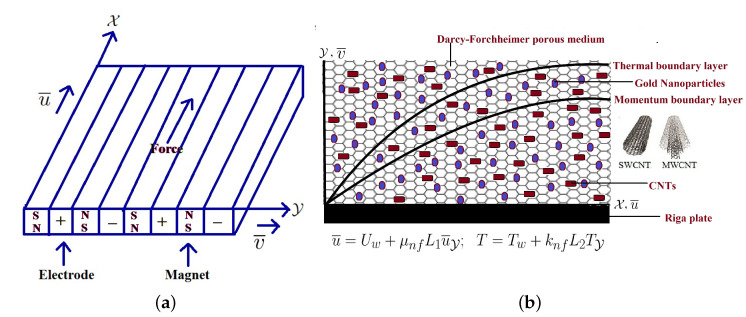
Riga plate (**a**) and physical configuration of the flow model (**b**).

The time-dependent DF flow of water-based CNTs/gold nanoparticles past a Riga plate is investigated. These components are assembled on a flat surface to make a Riga plate. Let $\bar{u}$ and $\bar{v}$ are the X and Y component velocities, respectively. The velocity and thermal slip impacts are included in our analysis. The SWCNTs (single-wall carbon nanotubes) and MWCNTs (multi-wall carbon nanotubes) are the two most-prevalent types of CNTs. In the energy expressions, the consequences of heat consumption are also taken into account. In energy expression, nonlinear radiation can be derived with the help of the nonlinear Rosseland approximation theory. Moreover, the free stream temperature and plate temperature are denoted as $T_{\infty }$ and $T_{w}$, respectively. The Riga plate and physical configuration of the flow model are displayed in Figure 1a,b. The mathematical modeling of continuity, momentum and energy expressions are defined in the following format (see Shafiq et al. [29], Ijaz Khan et al. [30] and Soomro et al. [31]):

(1) $$\begin{array}{ccc} & & {\bar{u}}_{X}+{\bar{v}}_{Y}=0 \end{array}$$

(2) $$\begin{array}{ccc} & & {\bar{u}}_{t}+\bar{u}{\bar{u}}_{X}+\bar{v}{\bar{u}}_{Y}=\nu_{nf}{\bar{u}}_{\mathrm{YY}}-\frac{\nu_{nf}}{k_{1}^{*}}\bar{u}-\frac{c_{b}}{\sqrt{k_{1}^{*}}}{\bar{u}}^{2}+\frac{\pi J_{0}M}{8\rho_{nf}}Exp\left[-\frac{\pi }{a_{1}}Y\right] \end{array}$$

(3) $$\begin{array}{ccc} & & T_{t}+\bar{u}T_{X}+\bar{v}T_{Y}=\alpha_{nf}T_{\mathrm{YY}}+\frac{16\sigma^{*}}{3k^{*}{\left(\rho c_{p}\right)}_{nf}}{\left(T^{3}T_{Y}\right)}_{Y}+\frac{Q}{{\left(\rho c_{p}\right)}_{nf}}\left(T-T_{\infty }\right) \end{array}$$

The corresponding constraints are

(4) $$\begin{array}{c} \bar{u}=U_{w}+\mu_{nf}L_{1}\bar{u}y;\;\bar{v}=0;\;T=T_{w}+k_{nf}L_{2}T_{Y}\;\mathrm{at}\;Y=0\\ \bar{u}\to 0;\bar{v}\to 0,T\to T_{\infty }\;\mathrm{as}\;Y\to \infty \end{array}$$

The description of each of the notations is offered in the nomenclature segment. Define (see Upadhya et al. [32])

(5) $$\begin{array}{ccc} & & \bar{u}=a{\left(1-\xi t\right)}^{-1}XF^{\prime }\left(℧\right);\;\;\;\bar{v}=-{\left(\frac{a\nu_{f}}{1-\xi t}\right)}^{\frac{1}{2}}F\left(℧\right);\;\;\;℧={\left(\frac{a}{\nu_{f}\left(1-\xi t\right)}\right)}^{\frac{1}{2}} \end{array}$$

Through the implementation of change (5) in expressions (2) and (3), the simplified expressions that are obtained are as follows:

(6) $$\begin{array}{c} \frac{A_{1}}{A_{2}}F^{\prime \prime \prime }\left(℧\right)-F^{\prime }{}^{2}\left(℧\right)+F^{\prime \prime }\left(℧\right)F\left(℧\right)-A\left[F^{\prime }\left(℧\right)+\frac{℧}{2}F^{\prime \prime }\left(℧\right)\right]-\frac{A_{1}}{A_{2}}\lambda F^{\prime }\left(℧\right)\\ -FrF^{\prime }{}^{2}\left(℧\right)+\frac{1}{A_{2}}\;HaExp\;\left[-\beta_{R}℧\right]=0 \end{array}$$

(7) $$\begin{array}{cc} \frac{A_{3}}{A_{4}} & \frac{1}{Pr}\theta^{\prime \prime }\left(℧\right)-F^{\prime }\left(℧\right)\theta \left(℧\right)+\theta^{\prime }\left(℧\right)F\left(℧\right)-A\left[\theta \left(℧\right)+\frac{℧}{2}\theta^{\prime }\left(℧\right)\right]+\frac{1}{A_{4}Pr}\frac{4}{3}R\left[{\left(\Lambda -1\right)}^{3}\right.\\ \; & \left\{3\theta^{2}\left(℧\right)\theta^{\prime }{}^{2}\left(℧\right)+\theta^{3}\left(℧\right)\theta^{\prime \prime }\left(℧\right)\right\}+{\left(\Lambda -1\right)}^{2}\left\{6\theta \left(℧\right)\theta^{\prime }{}^{2}\left(℧\right)+3\theta^{2}\left(℧\right)\theta^{\prime \prime }\left(℧\right)\right\}\\ \; & \left.+\left(\Lambda -1\right)\left\{3\theta^{\prime }{}^{2}\left(℧\right)+3\theta \left(℧\right)\theta^{\prime \prime }\left(℧\right)\right\}+\theta^{\prime \prime }\right]+\frac{1}{A_{4}}Hg\theta \left(℧\right)=0 \end{array}$$

The converted boundary constraints are

(8) $$\begin{array}{c} F\left(0\right)=0;\;F^{\prime }\left(0\right)=1+A_{1}K_{1}F^{\prime \prime }\left(0\right);\;F^{\prime }\left(\infty \right)=0;\\ \theta \left(0\right)=1+A_{3}K_{2}\theta^{\prime }\left(0\right);\;\theta \left(\infty \right)=0. \end{array}$$

An explanation of each non-dimensional number used in the preceding statements is provided in the nomenclature section.

## 3. Quantities of Physical Interest

### 3.1. Skin Friction Coefficient

The definition of the skin friction coefficient is expressed as

$$\begin{array}{c} C_{F}=\frac{2\tau_{XY}}{\rho_{nf}U_{w}^{2}} \end{array}$$

Here, $\tau_{XY}$ represents the wall shear stresses, which may be mathematically written as

$$\begin{array}{c} \tau_{XY}=\mu_{nf}{\left(\frac{\partial \bar{u}}{\partial Y}\right)}_{Y=0} \end{array}$$

The skin friction coefficient, in its dimensionless form, is written as

$$\begin{array}{c} \frac{1}{2}C_{F}\sqrt{Re}=A_{1}F^{\prime \prime }\left(0\right); \end{array}$$

### 3.2. Local Nusselt Number

The local Nusselt number is expressed as

$$\begin{array}{c} Nu=\frac{Xq_{w}}{k_{nf}\left(T_{f}-T_{\infty }\right)} \end{array}$$

where $q_{w}$ represents the amount of wall heat flux and is mathematically represented by

$$\begin{array}{c} q_{w}=-{\left[k_{nf}\left(\frac{\partial T}{\partial Y}\right)+\frac{16\sigma^{*}}{3k^{*}{\left(\rho c_{p}\right)}_{nf}}\frac{\partial }{\partial Y}\left(T^{3}\frac{\partial T}{\partial Y}\right)\right]}_{Y=0} \end{array}$$

The dimensionless representation of the local Nusselt number is formulated as

$$\begin{array}{c} \frac{Nu}{\sqrt{Re}}=-\left[A_{3}+\frac{4}{3}R{\left\{1+\theta (0)(\Lambda -1)\right\}}^{3}\right]\theta^{\prime }\left(0\right) \end{array}$$

## 4. Methodology

### 4.1. Analytical Method

The reduced mathematical expressions (6) and (7) with boundary constraints (8) are analytically computed by employing the HAM procedure. This approach is suitable for solving nonlinear problems analytically and provides a great degree of flexibility in picking starting estimates and linear operators for structuring solutions (see Zeeshan et al. [33]). As a result, we have:

Initial approximations:

$$\begin{array}{c} F_{0}\left(℧\right)=\frac{1}{1+K_{1}A_{1}}\left[1-\frac{1}{e^{℧}}\right];\;\;\theta_{0}\left(℧\right)=\frac{1}{\left(1+K_{1}A_{2}\right)e^{℧}} \end{array}$$

Linear operators:

$$\begin{array}{c} L_{F}=F^{\prime \prime \prime }-F^{\prime };\;\;L_{\theta }=\theta^{\prime \prime }-\theta \end{array}$$

Linear properties:

$$\begin{array}{c} L_{F}\left[\psi_{1}+\psi_{2}e^{℧}+\psi_{3}\frac{1}{e^{℧}}\right]=0=L_{\theta }\left[\psi_{4}e^{℧}+\psi_{5}\frac{1}{e^{℧}}\right] \end{array}$$

where $\psi_{i};i=1,2,\ldots ,5$ are constants.

Zeroth-order deformation problems:

$$\begin{array}{ccc} \left(1-Q\right)L_{F}\left[F\left(℧,Q\right)-F_{0}\left(℧\right)\right] & = & Qh_{F}R_{1}\left[F\left(℧,Q\right)\right]\\ \left(1-Q\right)L_{\theta }\left[\theta \left(℧,Q\right)-\theta_{0}\left(℧\right)\right] & = & Qh_{\theta }R_{2}\left[F\left(℧,Q\right),\theta \left(℧,Q\right)\right] \end{array}$$

Here, Q∈[0,1] is an embedding parameter and $R_{1}$ and $R_{2}$ are non-linear operators (see Khan et al. [34]).

The *n*th-order problems are:

$$\begin{array}{c} F_{n}\left(℧\right)=F_{n}^{*}\left(℧\right)+\psi_{1}+\psi_{2}e^{℧}+\psi_{3}\frac{1}{e^{℧}};\;\;\theta_{n}\left(℧\right)=\theta_{n}^{*}\left(℧\right)+\psi_{4}e^{℧}+\psi_{5}\frac{1}{e^{℧}}\;\; \end{array}$$

Here, $F_{n}^{*}\left(℧\right)$ and $\theta_{n}^{*}\left(℧\right)$ are the particular solutions.

**Figure 2:**
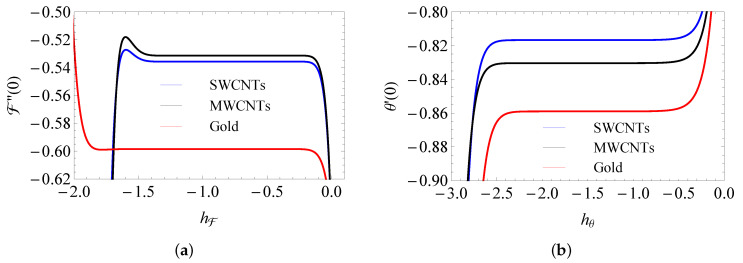
The h–curves of $F^{\prime \prime }(0)$ (**a**) and $\theta^{\prime }(0)$ (**b**).

The parameters $h_{F}$ and $h_{\theta }$ are the ones that take responsibility for the solution’s convergent implementation (see Prabakaran et al. [35] and Sajjad Haider et al. [36]). The ambit of $h_{F}$ is [−1.4, −0.2] (SWCNTs), [−1.4, −0.2] (MWCNTs), [−1.2, −0.38] (gold) and $h_{\theta }$ is [−2.4, −0.55] (SWCNTs), [−2.4, −0.55] (MWCNTs), [−2.3, −0.55] (gold); see Figure 2a,b. Mathematica software is used for all HAM calculations.

### 4.2. Numerical Method

The transferred nonlinear ODEs (6) and (7) with the boundary constraints under consideration (8) are numerically computed by implementing the MATLAB bvp4c solution procedure. In this context, we begin by transforming the system of higher-order ODEs into a system of first-order differential equations (see Ghani et al. [37] and Safak Kayikci et al. [38]).

Let $F=Z_{1},\;F^{\prime }=Z_{2},\;F^{\prime \prime }=Z_{3},\;\theta =Z_{4},\;\theta^{\prime }=Z_{5}$.

The system of equations is

$$\begin{array}{ccc} & & Z_{1}^{\prime }=Z_{2}\\ & & Z_{2}^{\prime }=Z_{3}\\ & & Z_{3}^{\prime }=\frac{Z_{2}^{2}-Z_{1}Z_{3}+A\left[Z_{2}+\frac{℧}{2}Z_{3}\right]+\frac{A_{1}}{A_{2}}\lambda Z_{2}-Ha\frac{1}{A_{2}}e^{-\beta_{R}℧}+FrZ_{2}^{2}}{\frac{A_{1}}{A_{2}}}\\ & & Z_{4}^{\prime }=Z_{5}\\ & & Z_{5}^{\prime }=\frac{E_{1}}{E_{2}} \end{array}$$

where

$$\begin{array}{ccc} & & E_{1}=Z_{2}Z_{4}-Z_{1}Z_{5}+A\left[Z_{4}+\frac{℧}{2}Z_{5}\right]-\frac{1}{A_{4}Pr}\frac{4}{3}R[{\left(\Lambda -1\right)}^{3}\left\{3Z_{4}^{2}Z_{5}^{2}\right\}+{\left(\Lambda -1\right)}^{2}\left\{6Z_{4}Z_{5}^{2}\right\}\\ & & \;\;+\left(\Lambda -1\right)\left\{3Z_{5}^{2}\right\}]-\frac{1}{A_{4}}HgZ_{4}\\ & & E_{2}=\frac{A_{3}}{A_{4}}\frac{1}{Pr}+\frac{1}{A_{4}Pr}\frac{4}{3}R\left[{\left(\Lambda -1\right)}^{3}\left\{Z_{4}^{3}\right\}+{\left(\Lambda -1\right)}^{2}\left\{3Z_{4}^{2}\right\}+\left(\Lambda -1\right)\left\{3Z_{4}\right\}+1\right] \end{array}$$

with the corresponding conditions

$$\begin{array}{ccc} & & Z_{1}\left(0\right)=0,\;Z_{2}\left(0\right)=1+A_{1}K_{1}Z_{3}\left(0\right),\;Z_{2}\left(\infty \right)=0,\\ & & Z_{4}\left(0\right)=1+A_{3}K_{2}Z_{5}\left(0\right),\;Z_{4}\left(\infty \right)=0, \end{array}$$

We solve the aforementioned problem by implementing the MATLAB bvp4c function with an error of $10^{-5}$ and a step size of 0.05.

## 5. Entropy Analysis

Entropy creation may be expressed mathematically as follows:

$$\begin{array}{ccc} S_{gen}= & & \frac{k_{f}}{T_{\infty }^{2}}\left[\frac{k_{nf}}{k_{f}}+\frac{16\sigma^{*}}{3k^{*}k_{f}}T^{3}\right]{\left(\frac{\partial T}{\partial y}\right)}^{2}+\frac{\mu_{nf}}{T_{\infty }}u^{2}+\frac{\mu_{nf}}{T_{\infty }}{\left(\frac{\partial u}{\partial y}\right)}^{2} \end{array}$$

The non-dimensional form of the entropy generation equation is

$$\begin{array}{ccc} N(℧)= & & Re\left(A_{3}+\frac{4}{3}R{\left[\theta (℧)(\Lambda -1)+1\right]}^{3}\right)\theta^{\prime }{}^{2}\left(℧\right)+A_{1}ReBr\frac{\lambda }{\alpha_{1}}F^{\prime }{}^{2}\left(℧\right)+A_{1}ReBr\frac{1}{\alpha_{1}}F^{\prime \prime }{}^{2}\left(℧\right) \end{array}$$

The Bejan number may be determined by taking the entire amount of entropy created and dividing it by the amount of entropy produced due to heat transfer.

$$\begin{array}{c} BE=\frac{Re\left(A_{3}+\frac{4}{3}R{\left[\theta (℧)(\Lambda -1)+1\right]}^{3}\right)\theta^{\prime }{}^{2}\left(℧\right)}{Re\left(A_{3}+\frac{4}{3}R{\left[\theta (℧)(\Lambda -1)+1\right]}^{3}\right)\theta^{\prime }{}^{2}\left(℧\right)+A_{1}ReBr\frac{\lambda }{\alpha_{1}}F^{\prime }{}^{2}\left(℧\right)+A_{1}ReBr\frac{1}{\alpha_{1}}F^{\prime \prime }{}^{2}\left(℧\right)} \end{array}$$

## 6. Results and Discussion

The purpose of this segment is to scrutinize the consequences of the various emerging parameters on nanofluid velocity, nanofluid temperature, skin friction coefficient, local Nusselt number, entropy profile and Bejan number. All graphical results are made by implementing the HAM method, and the bvp4c scheme is used only for comparative purposes. Table 1 represents the physical characteristics of CNTs, gold nanoparticles and water. The mathematical definitions of the thermophysical characteristics are expressed in Table 2. Table 3 presents the comparison of $-f^{\prime \prime }\left(0\right)$ with $A=Fr=\phi =Ha=K_{1}=0$ for different values of λ to the results of Akbara et al. [39] for different values of λ and are in good agreement. The HAM order of approximations of SWCNTs, MWCNTs and gold nanoparticles are demonstrated in Table 4. It is perceived that the 13th order is to be sufficient for all computations. Table 5 provides the analytical and numerical comparison of SFC for assorted estimates of *A*, $K_{1}$, Ha, λ, Fr and ϕ for SWCNTs, MWCNTs and gold nanoparticles. It is found that greater values *A*, λ, Fr and ϕ lead to decrease the surface shear stress for all cases. However, higher values of $K_{1}$ and Ha causes to increase the surface shear stress. In addition to this, the surface shear stress of MWCNTs is noticeably greater than that of SWCNTs and gold nanoparticles. The analytical and numerical computation of LNN for assorted estimates of *A*, *R*, Λ, $K_{2}$, Hg and ϕ for SWCNTs, MWCNTs and gold nanoparticles are described in Table 6. This table shows that the temperature gradient increases with higher values of *A*, *R* and Λ, and the opposite is attained for larger $K_{2}$, Hg and ϕ values.

**Figure 3:**
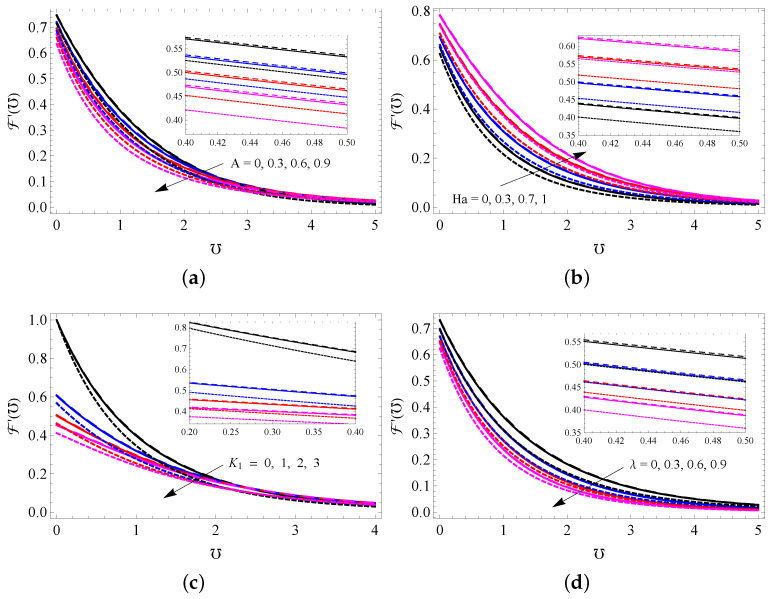
NF velocity profiles for varied assays of *A* (**a**), Ha (**b**), $K_{1}$ (**c**) and λ (**d**) for SWCNTs (solid line), MWCNTs (dashed line) and gold nanoparticles (dotted line).

**Figure 4:**
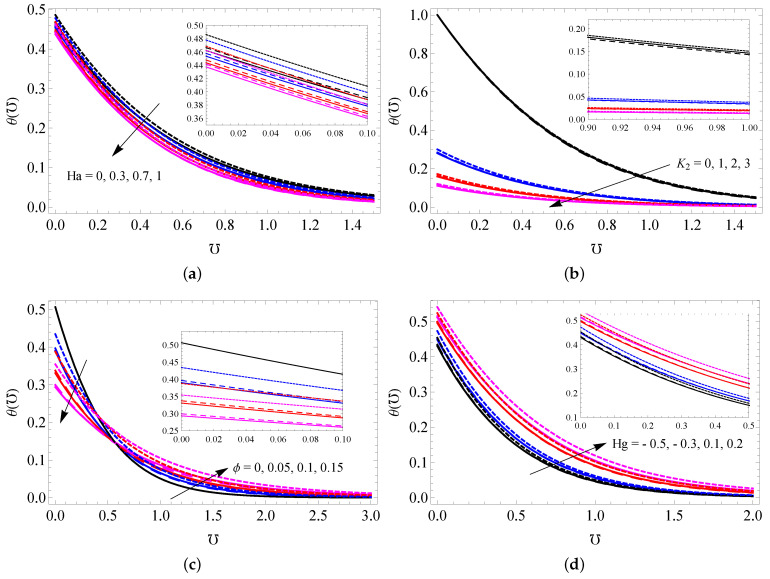
The NF temperature profile for varied assays of Ha (**a**), $K_{2}$ (**b**), ϕ (**c**) and Hg (**d**) for SWCNTs (solid line), MWCNTs (dashed line) and gold nanoparticles (dotted line).

**Figure 5:**
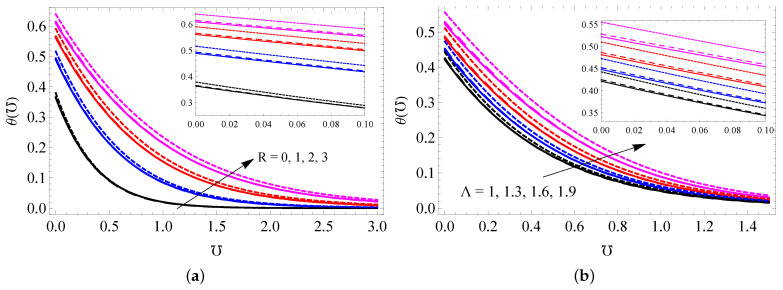
The NF temperature profile for varied assays of *R* (**a**) and Λ (**b**) for SWCNTs (solid line), MWCNTs (dashed line) and gold nanoparticles (dotted line).

Figure 3a–d show the impact of *A* (a), Ha (b), $K_{1}$ (c) and λ (d) on the NF velocity profile for all cases. It is seen that the NF velocity enhances when higher modified Hartmann number and it decreases for higher *A*, $K_{1}$ and λ values. Physically, a larger value for the porosity parameter creates more resistance to fluid flow, and this slow the fluid motion. A higher modified Hartmann number strengthens the external electric field, and this improves the fluid motion. Further, it is noted from these figures that MWCNTs have larger momentum boundary thickness compared to SWCNTs and gold nanoparticles because MWCNTs have low density values. The repercussions of Ha (a), $K_{2}$ (b), ϕ (c) and Hg (d) on the NF temperature profile are sketched in Figure 4a–d. It is noticed that upgrading the values of Hg increases the temperature of the nanofluid, but increasing the concentrations of Ha and $K_{2}$ has the opposite effect. NPVF causes the temperature of the nanofluid to decrease in the vicinity of the surface, while the temperature increases away from the surface. The responses of *R* and Λ on the temperature profile are captured in Figure 5a,b. Both *R* and Λ contribute to improving the nanofluid temperature. Physically, heat energy transport is improved because of higher values of the radiation parameter. Thus, the fluid becomes warmer and thicker the thermal boundary layer thickness.

**Figure 6:**
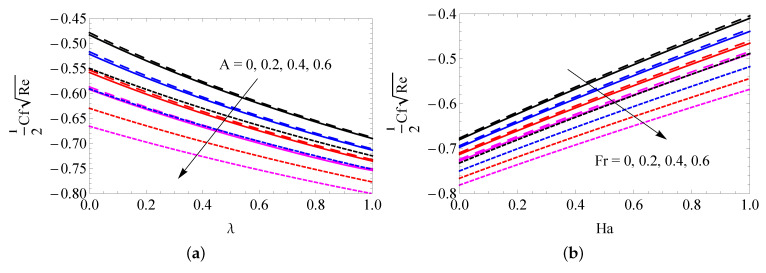
The changes to SFC versus *A* and λ (**a**) and Fr and Ha (**b**) for SWCNTs (solid line), MWCNTs (dashed line) and gold nanoparticles (dotted line).

**Figure 7:**
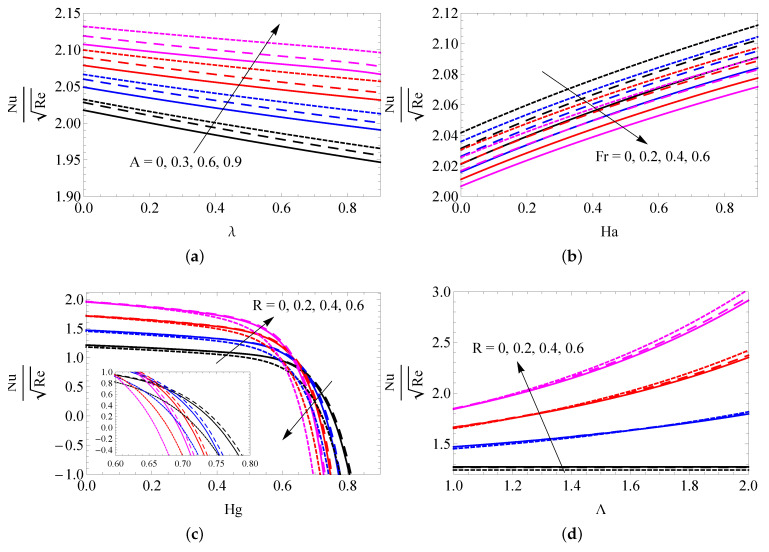
The changes to LNN versus *A* and λ (**a**), Fr and Ha (**b**), *R* and Hg (**c**) and *R* and Λ (**d**) for SWCNTs (solid line), MWCNTs (dashed line) and gold nanoparticles (dotted line).

**Figure 8:**
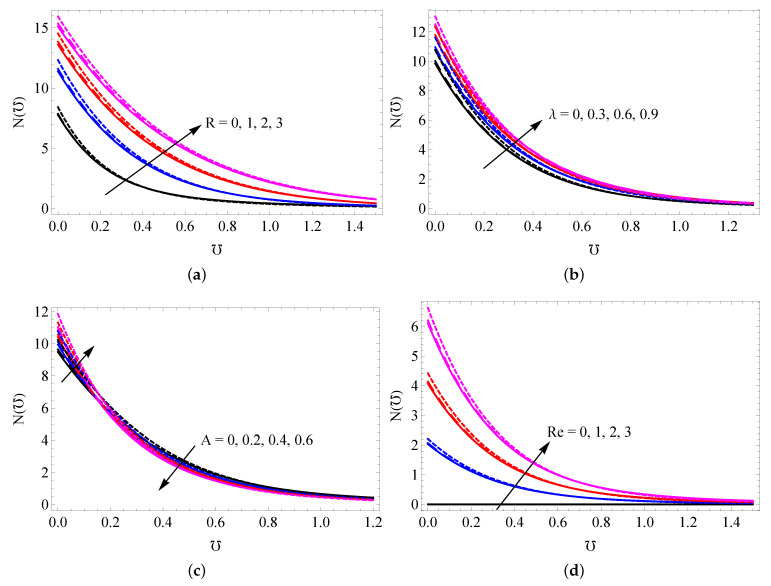
The changes to entropy profile versus *R* (**a**), λ (**b**), *A* (**c**) and Re (**d**) for SWCNTs (solid line), MWCNTs (dashed line) and gold nanoparticles (dotted line).

**Figure 9:**
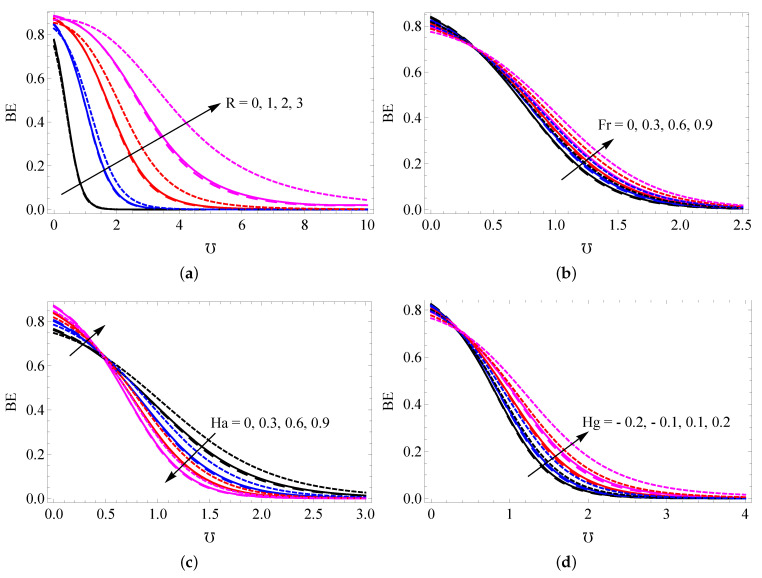
The changes to Bejan number versus *R* (**a**), Fr (**b**), Ha (**c**) and Hg (**d**) for SWCNTs (solid line), MWCNTs (dashed line) and gold nanoparticles (dotted line).

The changes to SFC versus *A*, λ, Fr and Ha for SWCNTs (solid line), MWCNTs (dashed line) and gold nanoparticles (dotted lines) are captured in Figure 6a,b. It is observed that the surface shear stress is decreased with increased values of *A*, λ and Fr, and the quite reverse trend occurs when increasing the Ha values. The changes to LNN versus *A* and λ (a), Fr and Ha (b), *R* and Hg (c) and *R* and Λ (d) are presented in Figure 7a–d. It is deduced that the temperature gradient increases with increasing *A* and Ha. LNN decreases with increased λ, Fr and Hg. The radiation parameter generates a higher heat transfer gradient when Hg<0.4 and its opposite behavior attains when Hg>0.4. The changes to the entropy profile versus *R* (a), λ (b), *A* (c) and Re (d) for SWCNTs (solid line), MWCNTs (dashed line) and gold nanoparticles (dotted line) were plotted in Figure 8a–d. It is ascertained that the entropy profile promotes when augmenting the values of *R*, λ and Re. The reverse trends were obtained in more quantity of *A*. Figure 9a–d elucidates the behavior of *R* (a), Fr (b), Ha (c) and Hg (d) on Bejan number. It is noted that the radiation parameter leads to improving the Bejan number. The Bejan number suppresses near the plate and improves away from the surface when improving the values of Fr and Hg. The reverse trend was obtained for changing the values of Ha.

**Figure 10:**
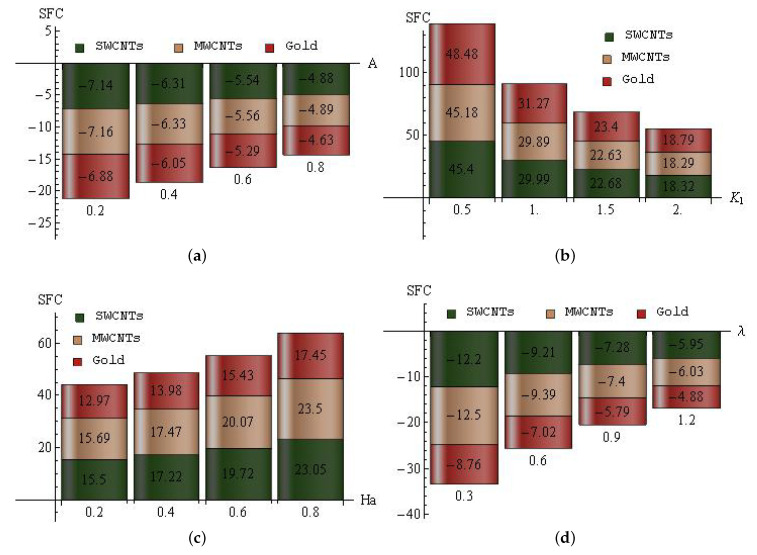
The increase/decrease percent of SFC for *A* (**a**), $K_{1}$ (**b**), Ha (**c**) and λ (**d**) for SWCNTs, MWCNTs and gold nanoparticles.

The increase/decrease percent of SFC for *A* (a), $K_{1}$ (b), Ha (c) and λ (d) for SWCNTs, MWCNTs and gold nanoparticles is displayed in Figure 10a–d. For unsteady parameter: In SWCNTs, the maximal decline percent (7.14) is attained when *A* is altered from 0 to 0.2, and the minimal decline percent (4.88) is attained when *A* is varied from 0.6 to 0.8. In MWCNTs, the maximal decline percent (7.16) is attained when *A* is altered from 0 to 0.2, and the minimal decline percent (4.89) is attained when *A* is varied from 0.6 to 0.8. In gold nanoparticles, the maximal decline percent (6.88) is attained when *A* is altered from 0 to 0.2, and the minimal decline percent (4.63) is attained when *A* is varied from 0.6 to 0.8; see Figure 10a. For slip parameter: In SWCNTs, the maximal ascent percent (45.4) is attained when $K_{1}$ is altered from 0 to 0.5, and the minimal ascent percent (18.32) is attained when $K_{1}$ is varied from 1.5 to 2. In MWCNTs, the maximal ascent percent (45.18) is attained when $K_{1}$ is altered from 0 to 0.5, and the minimal ascent percent (18.29) is attained when $K_{1}$ is varied from 1.5 to 2. In gold nanoparticles, the maximal ascent percent (48.48) is attained when $K_{1}$ is altered from 0 to 0.5, and the minimal ascent percent (18.79) is attained when $K_{1}$ is varied from 1.5 to 2; see Figure 10b. For the modified Hartmann number: In SWCNTs, the maximal ascent percent (23.05) is attained when Ha is altered from 0.6 to 0.8, and the minimal ascent percent (15.5) is attained when Ha is varied from 0 to 0.2. In MWCNTs, the maximal ascent percent (23.5) is attained when Ha is altered from 0.6 to 0.8, and the minimal ascent percent (15.69) is attained when Ha is varied from 0 to 0.2. In gold nanoparticles, the maximal ascent percent (17.45) is attained when Ha is altered from 0.6 to 0.8, and the minimal ascent percent (12.97) is attained when Ha is varied from 0 to 0.2; see Figure 10c. For porosity parameter: In SWCNTs, the maximal decline percent (12.2) is attained when λ is altered from 0 to 0.3, and the minimal decline percent (5.95) is attained when λ is varied from 0.9 to 1.2. In MWCNTs, the maximal decline percent (12.5) is attained when λ is altered from 0 to 0.3, and the minimal decline percent (6.03) is attained when λ is varied from 0.9 to 1.2. In gold nanoparticles, the maximal decline percent (8.76) is attained when λ is altered from 0 to 0.3, and the minimal decline percent (4.88) is attained when λ is varied from 0.9 to 1.2; see Figure 10d.

**Figure 11:**
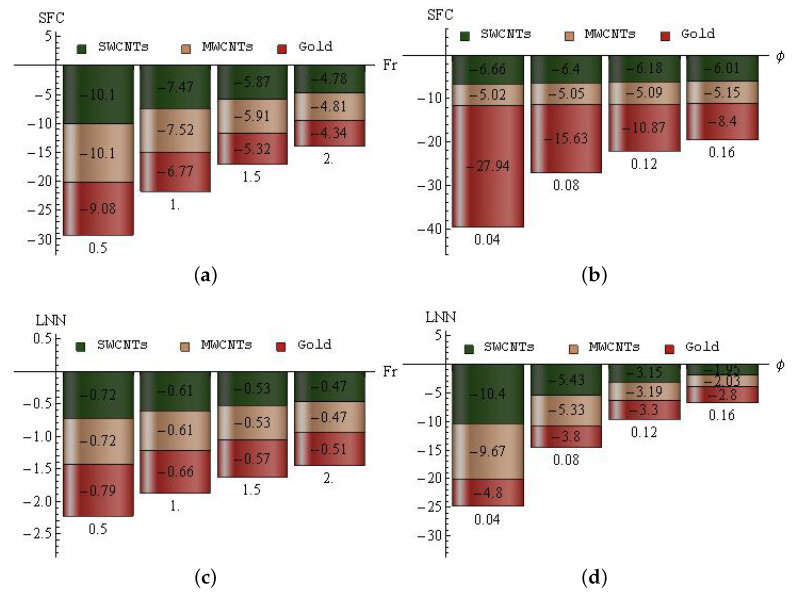
The decline percent of SFC for Fr (**a**) and ϕ (**b**) and LNN for Fr (**c**) and ϕ (**d**) for SWCNTs, MWCNTs and gold nanoparticles.

The decline percent of SFC for Fr (a) and ϕ (b) and LNN for Fr (c) and ϕ (d) for SWCNTs, MWCNTs and gold nanoparticles are plotted in Figure 11a–d. For the Forchheimer number: In SWCNTs, the maximal decline percent (10.1) is attained when Fr is altered from 0 to 0.5, and the minimal decline percent (4.78) is attained when Fr is varied from 1.5 to 2. In MWCNTs, the maximal decline percent (10.1) is attained when Fr is altered from 0 to 0.5, and the minimal decline percent (4.81) is attained when Fr is varied from 1.5 to 2. In gold nanoparticles, the maximal decline percent (9.08) is attained when Fr is altered from 0 to 0.5, and the minimal decline percent (4.34) is attained when Fr is varied from 1.5 to 2; see Figure 11a. For ϕ: In SWCNTs, the maximal decline percent (6.66) is attained when ϕ is altered from 0 to 0.04, and the minimal decline percent (6.01) is attained when ϕ is varied from 0.12 to 0.16. In MWCNTs, the maximal decline percent (5.15) is attained when ϕ is altered from 0.12 to 0.16, and the minimal decline percent (5.02) is attained when ϕ is varied from 0 to 0.04. In gold nanoparticles, the maximal decline percent (27.94) is attained when ϕ is altered from 0 to 0.04, and the minimal decline percent (8.4) is attained when ϕ is varied from 0.12 to 0.16; see Figure 11b. For the Forchheimer number: In SWCNTs, the maximal decline percent (0.72) is attained when Fr is altered from 0 to 0.5, and the minimal decline percent (0.47) is attained when Fr is varied from 1.5 to 2. In MWCNTs, the maximal decline percent (0.72) is attained when Fr is altered from 0 to 0.5, and the minimal decline percent (0.47) is attained when Fr is varied from 1.5 to 2. In gold nanoparticles, the maximal decline percent (0.79) is attained when Fr is altered from 0 to 0.5, and the minimal decline percent (0.51) is attained when Fr is varied from 1.5 to 2; see Figure 11c. For ϕ: In SWCNTs, the maximal decline percent (10.4) is attained when ϕ is altered from 0 to 0.04, and the minimal decline percent (1.95) is attained when ϕ is varied from 0.12 to 0.16. In MWCNTs, the maximal decline percent (9.67) is attained when ϕ is altered from 0 to 0.04, and the minimal decline percent (2.03) is attained when ϕ is varied from 0.12 to 0.16. In gold nanoparticles, the maximal decline percent (4.8) is attained when ϕ is altered from 0 to 0.04, and the minimal decline percent (2.8) is attained when ϕ is varied from 0.12 to 0.16; see Figure 11d.

**Figure 12:**
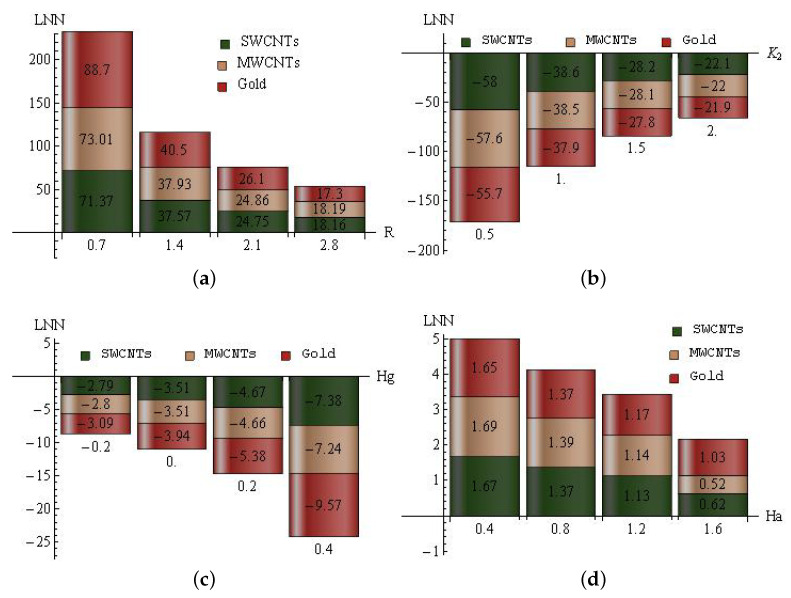
The increase/decrease percent of LNN for *R* (**a**), $K_{2}$(**b**), Hg (**c**) and Ha (**d**) for SWCNTs, MWCNTs and gold nanoparticles.

The increase/decrease percent of LNN for *R* (a), $K_{2}$ (b), Hg (c) and Ha (d) for SWCNTs, MWCNTs and gold nanoparticles are sketched in Figure 12a–d. For radiation parameter: In SWCNTs, the maximal ascent percent (71.37) is attained when *R* is altered from 0 to 0.7, and the minimal ascent percent (18.16) is attained when *R* is varied from 2.1 to 2.8. In MWCNTs, the maximal ascent percent (73.01) is attained when *R* is altered from 0 to 0.7, and the minimal ascent percent (18.19) is attained when *R* is varied from 2.1 to 2.8. In gold nanoparticles, the maximal ascent percent (88.7) is attained when *R* is altered from 0 to 0.7, and the minimal ascent percent (17.3) is attained when *R* is varied from 2.1 to 2.8; see Figure 12a. For slip parameter: In SWCNTs, the maximal decline percent (58) is attained when $K_{2}$ is altered from 0 to 0.5, and the minimal decline percent (22.1) is attained when $K_{2}$ is varied from 1.5 to 2. In MWCNTs, the maximal decline percent (57.6) is attained when $K_{2}$ is altered from 0 to 0.5, and the minimal decline percent (22) is attained when $K_{2}$ is varied from 1.5 to 2. In gold nanoparticles, the maximal decline percent (55.7) is attained when $K_{2}$ is altered from 0 to 0.5, and the minimal decline percent (21.9) is attained when $K_{2}$ is varied from 1.5 to 2; see Figure 12b. For heat consumption/generation parameter: In SWCNTs, the maximal decline percent (7.38) is attained when Hg is altered from 0.2 to 0.4, and the minimal decline percent (2.79) is attained when Hg is varied from −0.4 to −0.2. In MWCNTs, the maximal decline percent (7.24) is attained when Hg is altered from 0.2 to 0.4, and the minimal decline percent (2.8) is attained when Hg is varied from −0.4 to −0.2. In gold nanoparticles, the maximal decline percent (9.57) is attained when Hg is altered from 0.2 to 0.4, and the minimal decline percent (3.09) is attained when Hg is varied from −0.4 to −0.2; see Figure 12c. For the modified Hartann number: In SWCNTs, the maximal ascent percent (1.67) is attained when Ha is altered from 0 to 0.4, and the minimal ascent percent (0.62) is attained when Ha is varied from 1.2 to 1.6. In MWCNTs, the maximal ascent percent (1.69) is attained when Ha is altered from 0 to 0.4, and the minimal ascent percent (0.52) is attained when Ha is varied from 1.2 to 1.6. In gold nanoparticles, the maximal ascent percent (1.65) is attained when Ha is altered from 0 to 0.4, and the minimal ascent percent (1.03) is attained when Ha is varied from 1.2 to 1.6; see Figure 12d.

## 7. Conclusions

This current study explores the influence on time-dependent DF flow of water-based CNTs and gold nanoparticles across a Riga plate under various slip situations. In addition, nonlinear radiation as well as heat consumption are taken into consideration. The creation of entropy may be estimated via the use of a variety of flow parameters. The controlling mathematical flow models are remodeled into ODE equations using an appropriate transformation with symmetry variables. The simplified ODE models are solved analytically and numerically using the homotopy analysis method and the MATLAB bvp4c approach. The following is a summary of the significant findings of our study:

- Higher unsteady parameter, porosity and slip parameters reduce the velocity of the nanofluid.
- A higher-momentum boundary thickness occurs in MWCNTs compared to SWCNTs and gold nanoparticles because MWCNTs have low density values.
- The depth of the thermal boundary layer increases with increasing radiation, temperature ratio, and heat consumption/generation parameters.
- A higher Forchheimer number, unsteady parameter and porosity suppress the surface drag force.
- Radiation, unsteady parameter, temperature ratio and modified Hartmann number increase the temperature gradient values.
- More entropy is generated with higher radiation, porosity and Reynolds number.
- Increased radiation, heat consumption/generation and Forchheimer number increase the Bejan number.

## Figures and Tables

**Table 1:** Physical properties of CNTs, gold nanoparticles and water (see Hayat et al. [7] and Aman et al. [40]).

Physical Characteristics	SWCNTs	MWCNTs	Gold	Water
k (W/mK)	6600	3000	318	0.613
ρ (kg/m${}^{3}$)	2600	1600	19,300	997.1
$c_{p}\left(J/kgK\right)$	425	796	129	4179

**Table 2:** The mathematical definitions of the thermophysical characteristics are expressed as follows (see Cui et al. [41]).

Property	Symbol	Definition
Viscosity	$\mu_{nf}$	$\frac{\mu_{nf}}{\mu_{f}}=\frac{1}{{\left(1-\phi \right)}^{2.5}}=A_{1}$
Density	$\rho_{nf}$	$\frac{\rho_{nf}}{\rho_{f}}=\left(1-\phi +\phi \frac{\rho_{nf}}{\rho_{f}}\right)=A_{2}$
Thermal conductivity	$k_{nf}$	$Carbon\;nanotube:\frac{k_{nf}}{k_{f}}=\frac{\left(1-\phi \right)+2\phi \frac{k_{nf}}{k_{nf}-k_{f}}ln\frac{k_{nf}+k_{f}}{2k_{f}}}{\left(1-\phi \right)+2\phi \frac{k_{f}}{k_{nf}-k_{f}}ln\frac{k_{nf}+k_{f}}{2k_{f}}}=A_{3}$
		$Au\;nanoparticles:\frac{k_{nf}}{k_{f}}=\frac{k_{Au}+2k_{f}-2\phi \left(k_{f}-k_{Au}\right)}{k_{Au}+2k_{f}+\phi \left(k_{f}-k_{Au}\right)}=A_{3}$
Heat capacitance	${\left(\rho c_{p}\right)}_{nf}$	$\frac{{\left(\rho cp\right)}_{nf}}{{\left(\rho cp\right)}_{f}}=1-\phi +\phi \frac{{\left(\rho c_{p}\right)}_{nf}}{{\left(\rho c_{p}\right)}_{f}}=A_{4}$
Kinematic viscosity	$\nu_{nf}$	$\nu_{nf}=\frac{\mu_{nf}}{\rho_{nf}}$

**Table 3:** Comparison of $-f^{\prime \prime }\left(0\right)$ with $A=\lambda =Fr=\phi =Ha=K_{1}=0$ to the results of Akbara et al. [39].

λ	Present Study	Ref. [39]
0	1.0000	1.0000
1	1.4142	1.4132
5	2.4495	2.4485
10	3.3166	3.3165
100	10.0499	10.0498
500	22.3830	22.3831
1000	31.6386	31.6385

**Table 4:** Order of approximations of SWCNTs, MWCNTs and gold nanoparticles.

Order	$-f^{\prime \prime }\left(0\right)$	$-\theta^{\prime }\left(0\right)$	$-f^{\prime \prime }\left(0\right)$	$-\theta^{\prime }\left(0\right)$	$-f^{\prime \prime }\left(0\right)$	$-\theta^{\prime }\left(0\right)$
1	0.52227	0.78899	0.51592	0.79964	0.60070	0.89490
5	0.53554	0.81645	0.53184	0.82653	0.59856	0.93767
10	0.53568	0.81673	0.53136	0.83040	0.59853	0.93778
13	0.53568	0.81672	0.53136	0.83039	0.59853	0.93779
15	0.53568	0.81672	0.53136	0.83039	0.59853	0.93779
20	0.53568	0.81672	0.53136	0.83039	0.59853	0.93779

**Table 5:** The analytical and numerical comparison of SFC for assorted estimates of *A*, $K_{1}$, Ha, λ, Fr and ϕ for SWCNTs, MWCNTs and gold nanoparticles.

						SWCNTs		MWCNTs		Gold	
A	$K_{1}$	Ha	λ	Fr	ϕ	***HAM***	NM	***HAM***	NM	***HAM***	NM
0	0.5	0.5	0.1	0.2	0.02	−0.50957	−0.50957	−0.50532	−0.50532	−0.57142	−0.57142
0.2						−0.54595	−0.54595	−0.54149	−0.54149	−0.61072	−0.61072
0.4						−0.58038	−0.58038	−0.57575	−0.57575	−0.64769	−0.64769
0.6						−0.61254	−0.61254	−0.60776	−0.60776	−0.68193	−0.68193
0.3	0	0.5	0.1	0.2	0.02	−1.03188	−1.03188	−1.01956	−1.01956	−1.22202	−1.22202
	0.5					−0.56344	−0.56344	−0.55889	−0.55889	−0.62953	−0.62953
	1					−0.39449	−0.39449	−0.39181	−0.39181	−0.43269	−0.43269
	1.5					−0.30501	−0.30502	−0.30314	−0.30315	−0.33142	−0.33142
0.3	0.5	0	0.1	0.2	0.02	−0.69748	−0.69748	−0.69390	−0.69390	−0.75011	−0.75010
		0.4				−0.58936	−0.58936	−0.58500	−0.58500	−0.65285	−0.65285
		0.8				−0.48787	−0.48787	−0.48278	−0.48278	−0.56157	−0.56157
		1.2				−0.39166	−0.39154	−0.38589	−0.38574	−0.47494	−0.47494
0.3	0.5	0.5	0	0.2	0.02	−0.54015	−0.54015	−0.53525	−0.53525	−0.61082	−0.61082
			0.3			−0.60605	−0.60605	−0.60210	−0.60209	−0.66432	−0.66432
			0.6			−0.66189	−0.66189	−0.65861	−0.65861	−0.71094	−0.71094
			0.9			−0.71010	−0.71010	−0.70733	−0.70733	−0.75209	−0.75209
0.3	0.5	0.5	0.1	0	0.02	−0.54025	−0.54025	−0.53573	−0.53573	−0.60600	−0.60600
				0.5		−0.59458	−0.59458	−0.59000	−0.59000	−0.661039	−0.661039
				1		−0.63901	−0.63901	−0.63439	−0.63439	−0.70577	−0.70577
				1.5		−0.67650	−0.67650	−0.67186	−0.67186	−0.74333	−0.74333
0.3	0.5	0.5	0.1	0.2	0	−0.54537	−0.54537	−0.54537	−0.54537	−0.54537	−0.54537
					0.04	−0.58170	−0.58170	−0.57277	−0.57277	−0.69776	−0.69776
					0.08	−0.61891	−0.61891	−0.60170	−0.60170	−0.80683	−0.80684
					0.12	−0.65718	−0.65718	−0.63235	−0.63235	−0.89457	−0.89457

**Table 6:** The analytical and numerical comparison of LNN for assorted estimates of *A*, *R*, Λ, $K_{2}$, Hg and ϕ for SWCNTs, MWCNTs and gold nanoparticles.

						SWCNTs		MWCNTs		Gold	
A	R	**Λ**	$K_{2}$	Hg	ϕ	***HAM***	NM	***HAM***	NM	***HAM***	NM
0	0.6	1.3	0.5	−0.3	0.02	2.00971	2.00939	2.02019	2.01981	2.08009	2.08035
0.2						2.04159	2.04158	2.05232	2.05232	2.11918	2.11918
0.4						2.07263	2.07263	2.08368	2.08368	2.15686	2.15686
0.6						2.10263	2.10249	2.11406	2.11384	2.19310	2.19325
0.3	0	1.3	0.5	−0.3	0.02	1.27290	1.27292	1.26844	1.26846	1.20932	1.20908
	0.7					2.18143	2.18143	2.19451	2.19451	2.28142	2.28142
	1.4					3.00092	3.00092	3.02698	3.02698	3.20457	3.20457
	2.1					3.74364	3.74364	3.77955	3.77955	4.01436	4.01435
0.3	0.6	1	0.5	−0.3	0.02	1.84144	1.84144	1.84705	1.84705	1.86722	1.86722
		1.3				2.05725	2.05725	2.06814	2.06814	2.13817	2.13817
		1.6				2.35097	2.35097	2.36957	2.36957	2.51232	2.51233
		1.9				2.75212	2.75212	2.78149	2.78149	3.02150	3.02150
0.3	0.6	1.3	0	−0.3	0.02	4.89983	4.89983	4.88272	4.88272	4.54935	4.54935
			0.5			2.05725	2.05725	2.06814	2.06814	2.13817	2.13817
			1			1.26255	1.26255	1.27158	1.27158	1.34872	1.34872
			1.5			0.90693	0.90693	0.91403	0.91403	0.97904	0.97904
0.3	0.6	1.3	0.5	−0.4		2.08498	2.08498	2.09610	2.09610	2.17191	2.17191
				−0.2		2.0268	2.02685	2.03750	2.03750	2.10112	2.10112
				0		1.95570	1.95570	1.96594	1.96594	2.01415	2.01415
				0.2		1.86433	1.86433	1.87437	1.87436	1.90130	1.90135
				0.4		1.72674	1.72674	1.73861	1.73852	1.72127	1.72121
0.3	0.6	1.3	0.5	−0.3	0	2.19454	2.19454	2.19454	2.19454	2.19454	2.19454
					0.04	1.96740	1.96740	1.98241	1.98241	2.08984	2.08984
					0.08	1.86052	1.86052	1.87681	1.87681	2.00944	2.00944
					0.12	1.80187	1.80187	1.81687	1.81687	1.94409	1.94409

## Data Availability

Not applicable.

## Nomenclature

**Symbols**	**Description**
$a,a_{1}$	positive constant
*A*	unsteady parameter
$\alpha =\frac{k}{\rho c_{p}}$	thermal diffusivity
$\alpha_{1}$	temperature difference parameter
Br	Brinkman number
$\beta_{R}$	dimensionless parameter
$c_{b}$	drag coefficient
$c_{p}$	specific heat
$\frac{1}{2}C_{F}\sqrt{Re}$	skin friction coefficient
f,nf	subscript represents the base fluid and the carbon nanotube/nanofluid
Fr	Forchheimer number
Ha	modified Hartmann number
Hg	heat consumption/generation parameter
$J_{0}$	current density applied to the electrodes
$k_{1}{}^{*}$	permeability of the porous medium
$K_{1}$	velocity slip parameter
$K_{2}$	temperature slip parameter
$k^{*}$	coefficient of mean absorption
$L_{1}$	velocity slip factor
$L_{2}$	temperature slip factor
λ	porosity parameter
Λ	temperature ratio parameter
*M*	is the magnetic property of the permanent magnets that are organized on top of the plate surface
℧	dimensionless variable
μ	dynamic viscosity
ν	kinematic viscosity
$\frac{Nu}{\sqrt{Re}}$	local Nusselt number
Pr	Prandtl number
*Q*	heat generation or absorption coefficient
*R*	radiation parameter
Re	Reynolds number
ρ	density
$\sigma^{*}$	Stefan-Boltzmann constant
*T*	temperature of the fluid
$T_{w}$	surface temperature
$T_{\infty }$	ambient temperature
θ	dimensionless temperature
$\bar{u},\bar{v}$	velocity components
X,Y	Cartesian coordinates
**Abbreviations**	**Explanation**
CNTs	carbon nanotubes
DF	Darcy-Forchheimer
HAM	homotopy analysis method
HT	heat transfer
LNN	local Nusselt number
MHD	magnetohydrodynamics
MWCNTs	multi-wall carbon nanotubes
NF	nanofluid
NM	numerical method
NPVF	nanoparticle volume fraction
ODE	ordinary differential equation
SFC	skin friction coefficient
SS	stretching sheet/surface
SWCNTs	single-wall carbon nanotubes

## References

[1] Saleh H., Alali E., Ebaid A. (2017). Medical applications for the flow of carbon-nanotubes suspended nanofluids in the presence of convective condition using Laplace transform. J. Assoc. Arab Univ. Basic Appl. Sci..

[2] Anuar N.S., Bachok N., Arifin N.M., Rosali H. (2020). Role of multiple solutions in flow of nanofluids with carbon nanotubes over a vertical permeable moving plate. Alex. Eng. J..

[3] Hayat T., Haider F., Muhammad T., Alsaedi A. (2019). Numerical treatment for Darcy-Forchheimer flow of carbon nanotubes due to an exponentially stretching curved surface. J. Cent. South Univ..

[4] Kamali R., Binesh A. (2010). Numerical investigation of heat transfer enhancement using carbon nanotube-based non-Newtonian nanofluids. Int. Commun. Heat Mass Transf..

[5] Saeed F.R., Jasim M.A., Mahmood N.B., Jaffar Z.M. (2021). Numerical investigation of convective heat transfer of single wall carbon nanotube nanofluid laminar flow inside a circular tube. Arch. Thermodyn..

[6] Hussain S.T., Haq R.U., Khan Z.H., Nadeem S. (2016). Water driven flow of carbon nanotubes in a rotating channel. J. Mol. Liq..

[7] Hayat T., Haider F., Muhammad T., Alsaedi A. (2017). Three-dimensional rotating flow of carbon nanotubes with Darcy-Forchheimer porous medium. PLoS ONE.

[8] Ghani S.N.A., Yarmand H., Noor N.F.M. (2022). Assorted graphene-based nanofluid flows near a reversed stagnation point over an inclined permeable cylinder. Proc. Natl. Acad. Sci. India Sect. A Phys. Sci..

[9] Rasool G., Zhang T., Shafiq A. (2019). Second-grade nano-fluidic flow past a convectively heated vertical Riga plate. Phys. Scr..

[10] Vishnu Ganesh N., Al-Mdalla Q.M., Al Fahel S., Dadoa S. (2019). Riga-Plate flow of *γAl*_2_*O*_3_-water/ethylene glycol with effective Prandtl number impacts. Heliyon.

[11] Abbas N., Nadeem S., Saleem S., Issakhov A. (2021). Transportation of modified nanofluid flow with time dependent viscosity over a Riga plate: Exponentially stretching. Ain Shams Eng. J..

[12] Vaidya H., Prasad K.V., Tlili I., Makinde O.D., Rajashekhar C., Khan S.U., Kumar R., Mahendra D.L. (2021). Mixed convective nanofluid flow over a non linearly stretched Riga plate. Case Stud. Therm. Eng..

[13] Ramesh G.K., Roopa G.S., Gireesha B.J., Shehzad S.A., Abbasi F.M. (2017). An electro-magneto-hydrodynamic flow Maxwell nanoliquid past a Riga plate: A numerical study. J. Braz. Soc. Mech. Sci. Eng..

[14] Madhukesh J.K., Ramesh G.K., Aly E.H., Chamkha A.J. (2022). Dynamics of water conveying SWCNT nanoparticles and swimming microorganisms over a Riga plate subject to heat source/sink. Alex. Eng. J..

[15] Waqas H., Kafait A., Muhammad T., Farooq U. (2022). Numerical study for bio-convection flow of tangent hyperbolic nanofluid over a Riga plate with activation energy. Alex. Eng. J..

[16] Ghasemi S.E., Mohsenian S., Gouran S., Zolfagharian A. (2022). A novel spectral relaxation approach for nanofluid flow past a stretching surface in presence of magnetic field and nonlinear radiation. Results Phys..

[17] Abdul Hakeem A.K., Ragupathi P., Saranya S., Ganga B. (2020). Three dimensional non-linear radiative nanofluid flow over a Riga plate. J. Appl. Comput. Mech..

[18] Gautam A.K., Rajput S., Bhattacharyya K., Pandey A.K., Chamkha A.J., Begum M. (2022). Comparative study of two non-Newtonian fluids with bioconvective induced MHD flow in presence of multiple slips, heat source/sink and nonlinear thermal radiation. Chem. Eng. J. Adv..

[19] Rashid A., Ayaz M., Islam S., Saeed A., Kumam P., Suttiarporn P. (2022). Theoretical analysis of the MHD flow of a tangent hyperbolic hybrid nanofluid over a stretching sheet with convective conditions: A nonlinear thermal radiation case. S. Afr. J. Chem. Eng..

[20] Eswaramoorthi S., Loganathan K., Reema J., Sonam G. (2022). Darcy-Forchheimer 3D flow of glycerin-based carbon nanotubes on a Riga plate with nonlinear thermal radiation and Cattaneo-Christov heat flux. J. Nanomater..

[21] Bejan A. (1979). A study of entropy generation in fundamental convective heat transfer. J. Heat Transf..

[22] Bejan A. (1982). Entropy Generation Through Heat and Fluid Flow.

[23] Eswaramoorthi S., Divya S., Muhammad F., Ngawang N. (2022). Entropy and heat transfer analysis for MHD flow of Cu/Ag-water-based nanofluid on a heated 3D plate with nonlinear radiation. Math. Probl. Eng..

[24] Dadheech P.K., Agrawal P., Sharma A., Dadheech A., Al-Mdallal Q., Purohit S.D. (2021). Entropy analysis for radiative inclined MHD slip flow with heat source in porous medium for two different fluids. Case Stud. Therm. Eng..

[25] Srinivas Reedy C., Srihari P., Ali F., Naikoti F. (2022). Numerical analysis of Carreau fluid flow over a vertical porous microchannel with entropy generation. Partial. Differ. Equ. Appl. Math..

[26] Abbas Z., Naveed M., Hussain M., Salamat N. (2020). Analysis of entropy generation for MHD flow of viscous fluid embedded in a vertical porous channel with thermal radiation. Alex. Eng. J..

[27] Ibrahim W., Gamachu D., Bedada B. (2022). Entropy generation analysis of three dimensional mixed convection flow of couple stress nanofluid with non-Fourier’s heat and non-Fick’s mass flux model. Alex. Eng. J..

[28] Khan M.I., Alzahrani F., Hobiny A., Ali Z. (2020). Estimation of entropy generation in Carreau-Yasuda fluid flow using chemical reaction with activation energy. J. Mater. Res. Technol..

[29] Shafiq A., Zari I., Khan I., Khan T.S. (2020). Marangoni driven boundary layer flow of carbon nanotubes towards a Riga plate. Front. Phys..

[30] Khan M.I., Qayyum S., Shah F., Kumar R.N., Gowda R.P., Prasannakumara B.C., Chu Y.M., Kadry S. (2021). Marangoni convective flow of hybrid nanofluid (*MnZnFe*_2_*O*_4_-*NiZnFe*_2_*O*_4_-*H*_2_*O*) with Darcy Forchheimer medium. Ain Shams Eng. J..

[31] Soomro F.A., Haq R.U., Al-Mdallal Q.M., Zhang Q. (2018). Heat generation/absorption and nonlinear radiation effects on stagnation point flow of nanofluid along a moving surface. Results Phys..

[32] Upadhya S.M., Raju C.S.K., Mahesha S. (2018). Nonlinear unsteady convection on micro and nanofluids with Cattaneo-Christov heat flux. Results Phys..

[33] Zeeshan A., Shehzad N., Atif M., Ellahi R., Sait S.M. (2022). Electromagnetic flow of SWCNT/MWCNT suspensions in two immiscible water- and engine-oil-based Newtonian fluids through porous media. Symmetry.

[34] Khan A., Jamshed W., Eid M.R., Pasha A.A., Tag El Din E.S.M., El-Wahed Khalifa H.A., Alharbi S.K. (2022). Unsteady electro-hydrodynamic stagnating point flow of hybridized nanofluid via a convectively heated enlarging (dwindling) surface with velocity slippage and heat generation. Symmetry.

[35] Prabakaran R., Eswaramoorthi S., Loganathan K., Sarris I.E. (2022). Investigation on thermally radiative mixed convective flow of carbon nanotubes/*Al*_2_*O*_3_ nanofluid in water past a stretching plate with joule heating and viscous dissipation. Micromachines.

[36] Haider S., Butt A.S., Li Y.Z., Imran S.M., Ahmad B., Tayyaba A. (2020). Study of entropy generation with multi-slip effects in MHD unsteady flow of viscous fluid past an exponentially stretching surface. Symmetry.

[37] Ghani S.N.A., Yarmand H., Noor N.F.M. (2021). Graphene-based Newtonian nanoliquid flows over an inclined permeable moving cylinder due to thermal stratification. Therm. Sci..

[38] Kayikci S., Eswaramoorthi S., Postalcioglu S., Loganathan K. (2022). Thermal analysis of radiative water-and glycerin-based carbon nanotubes past a Riga plate with stratification and non-Fourier heat flux theory. J. Therm. Anal. Calorim..

[39] Akbara N.S., Tripathib D., Khanc Z.H. (2018). Numerical investigation of Cattanneo-Christov heat flux in CNT suspended nanofluid flow over a stretching porous surface with suction and injection. Discret. Contin. Dyn. Syst.-S.

[40] Aman S., Khan I., Ismail Z., Salleh M.Z. (2018). Impacts of gold nanoparticles on MHD mixed convection Poiseuille flow of nanofluid passing through a porous medium in the presence of thermal radiation, thermal diffusion and chemical reaction. Neural Comput. Applic..

[41] Cui J., Jan A., Farooq U., Hussain M., Khan W.A. (2022). Thermal analysis of radiative Darcy–Forchheimer nanofluid flow across an inclined stretching surface. Nanomaterials.

